# Development of an ultra-short measure of eight domains of health-related quality of life for research and clinical care: the patient-reported outcomes measurement information system® PROMIS®-16 profile

**DOI:** 10.1007/s11136-023-03597-6

**Published:** 2024-02-06

**Authors:** Maria Orlando Edelen, Chengbo Zeng, Ron D. Hays, Anthony Rodriguez, Janel Hanmer, Judy Baumhauer, David Cella, Bryce B. Reeve, Patricia M. Herman

**Affiliations:** 1 Patient Reported Outcomes, Value and Experience (PROVE) Center, Department of Surgery, Brigham and Women’s Hospital, Boston, MA USA; 2 RAND Corporation, Behavioral and Policy Sciences, 20 Park Plaza #920, Boston, MA USA; 3 Division of General Internal Medicine and Health Services Research, UCLA Department of Medicine, Los Angeles, CA USA; 4 Division of General Internal Medicine, University of Pittsburgh, Pittsburgh, PA USA; 5 School of Medicine and Dentistry, University of Rochester, Rochester, NY USA; 6 Feinberg School of Medicine, Northwestern University, Chicago, IL USA; 7 Department of Population Health Sciences, Duke University School of Medicine, Durham, NC USA; 8 Behavioral and Policy Sciences, RAND Corporation, 1776 Main Street, Santa Monica, CA USA

**Keywords:** PROMIS profile, Health-related quality of life, Short-form development, Clinical screening

## Abstract

**Purpose:**

We describe development of a short health-related quality of life measure, the patient-reported outcomes measurement information system® (PROMIS®)-16 Profile, which generates domain-specific scores for physical function, ability to participate in social roles and activities, anxiety, depression, sleep disturbance, pain interference, cognitive function, and fatigue.

**Methods:**

An empirical evaluation of 50 candidate PROMIS items and item pairs was conducted using data from a sample of 5775 respondents from Amazon’s Mechanical Turk (MTurk). Results and item response theory information curves for a subset of item pairs were presented and discussed in a stakeholder meeting to narrow the candidate item sets. A survey of the stakeholders and 124 MTurk adults was conducted to solicit preferences among remaining candidate items and finalize the measure.

**Results:**

Empirical evaluation showed minimal differences in basic descriptive statistics (e.g., means, correlations) and associations with the PROMIS-29 + 2 Profile, thus item pairs were further considered primarily based on item properties and content. Stakeholders discussed and identified subsets of candidate item pairs for six domains, and final item pairs were agreed upon for two domains. Final items were selected based on stakeholder and MTurk-respondent preferences. The PROMIS-16 profile generates eight domain scores with strong psychometric properties.

**Conclusion:**

The PROMIS-16 Profile provides an attractive brief measure of eight distinct domains of health-related quality of life, representing an ideal screening tool for clinical care, which can help clinicians quickly identify distinct areas of concern that may require further assessment and follow-up. Further research is needed to confirm and extend these findings.

**Supplementary Information:**

The online version contains supplementary material available at 10.1007/s11136-023-03597-6.

## Plain English summary

The patient-reported outcomes measurement information system (PROMIS) 16-item Profile (PROMIS-16) was developed to be minimally burdensome, clinically useful, and able to generate eight health-related quality of life domain-specific scores (physical function, ability to participate in social roles and activities, anxiety, depression, sleep disturbance, pain interference, cognitive function, and fatigue). The PROMIS-16 was developed in three phases. In the first phase, a thorough empirical evaluation of all candidate PROMIS items and item pairs was conducted using data from a sample of adults from Amazon’s Mechanical Turk (MTurk) panel. This included basic descriptive information and associations with the PROMIS-29 + 2 Profile. In the second phase, a stakeholder meeting was held to discuss the findings. Final item pairs were agreed upon for two domains, and the candidate sets for the remaining domains were reduced. In the third phase, a survey of the stakeholder panel and another sample of MTurk adults was conducted to solicit preferences for one of two remaining item pairs for each of the other six domains. Stakeholders and MTurk respondents had similar preferences among the remaining candidate item pairs, and final items were selected based on those preferences. The results of the development process showed that the PROMIS-16 has good psychometric properties. The PROMIS-16 is a promising new brief measure of eight distinct domains of health-related quality of life for clinical care and research, representing an ideal screening tool for clinical care, which can help clinicians quickly identify distinct areas of concern that may require further assessment and follow-up. Further research is needed to confirm these findings and to evaluate the PROMIS-16 Profile in real-world settings.

## Introduction

The patient-reported outcomes measurement information system® (PROMIS®) [1, 2] includes an extensive portfolio of health-related quality of life (HRQOL) measures that are used around the world in research- and practice-based settings due to their psychometric soundness, flexibility of administration, and scoring normed to the United States general population. The PROMIS library has domain-specific (e.g., anxiety, pain) and global (e.g., general health) measures and offers a collection of pre-packaged multiple-domain measures called PROMIS Profiles (PROMIS-29, -43, -57) [3] that yield seven domain scores: anxiety, depression, fatigue, pain interference, physical function, sleep disturbance, and ability to participate in social roles and activities. The domain scores can be aggregated into physical and mental health summary scores [4] and six of them (anxiety is not included) plus a PROMIS measure of cognitive function can be used to calculate the PROMIS-based preference score (PROPr) [5, 6].

The PROMIS Profiles have seen a rapid uptake in health research settings given their accessibility, ability to describe in detail HRQOL domains that are specific and actionable, and summary scores. However, despite the push to implement clinically relevant patient-reported outcome data collection in clinical care, even the shortest PROMIS Profile, the PROMIS-29, may be considered too burdensome for routine clinical use and some research settings, leading many to decide not to measure HRQOL or opt for the more feasible PROMIS Global-10 [7]. The Global-10, a brief general measure that provides mental and physical health summary scores, is particularly useful for general surveillance and risk adjustment but it does not provide clinically actionable HRQOL domain-specific scores (e.g., pain interference score, depression score).

Thus, although PROMIS offers a host of measurement options, it does not provide an off-the-shelf domain profile option that is regarded as sufficiently brief for routine clinical use. In this article, we describe the development and provide evidence for the reliability and validity of a short PROMIS profile measure that represents eight HRQOL domains (physical function, ability to participate in social roles and activities, anxiety, depression, sleep disturbance, pain interference, cognitive function, and fatigue) with two items each: the PROMIS-16 Profile.

## Methods

### Participants

#### Amazon’s mechanical turk (MTurk) development sample

We collected demographic, clinical, and PROMIS item-level data (described further below) for this study as part of a larger survey from MTurk participants that used the online platform CloudResearch (formerly TurkPrime) to collect the data in 2021 [8]. Eligible study participants were 18 years or older with an IP address in the USA and had to have completed a minimum of 500 previous MTurk “human intelligence tasks” (surveys, writing product descriptions, coding, or identifying content in images or videos) with a successful completion rate of at least 95%. The 95% threshold was selected because it is associated with better response quality [9]. Additional quality control measures included deploying small batches of surveys hourly over several weeks to reduce selection bias, screening for excessive speediness in completing the survey (< 1 s per item) ,and including two fake conditions in a list of chronic health conditions [10].

All MTurk participants provided electronic consent at the start of the survey and were paid $1.50, an amount based on the expected time needed to complete the survey and the US federal minimum wage. Of the 6997 respondents who enrolled in the survey, 247 were excluded because they did not complete the survey, and 975 were excluded based on endorsing a fake condition. The final analytic sample of 5775 respondents had a median age of 37 years, was predominantly White (82%), non-Hispanic (86%), male (53%), and well-educated (over 65% had a bachelor’s degree or higher). Rates of endorsement for chronic conditions ranged from 4% (stroke) to 40% (back pain; see Table 1).

**Table 1:** Demographic characteristics of participants in MTurk development (N = 5775) and preference (N = 124) samples

Characteristic	Development sample	Preference sample
Age (median, IQR)	37 (31, 47)	37 (31, 44)
	N (%)	N (%)
Race		
White	4699 (82.3)	96 (82.8)
Black or African American	660 (11.6)	13 (11.2)
Asian or Asian American	409 (7.2)	4 (4.5)
White; Asian	0 (0.0)	1 (0.9)
White Black or African American	0 (0.0)	1 (0.9)
Native Hawaiian or Pacific Islander	36 (0.6)	1 (0.9)
Native American	39 (0.7)	0 (0.0)
Other races	44 (0.8)	0 (0.0)
Multiracial	151 (2.7)	0 (0.0)
Ethnicity		
Non-Hispanic	4902 (85.8)	112 (94.9)
Hispanic	812 (14.2)	6 (5.1)
Sex		
Female	2617 (45.8)	42 (35.9)
Male	3047 (53.3)	74 (63.3)
Transgender	28 (0.5)	1 (0.9)
Do not identify as female, male, or transgender	23 (0.4)	0 (0.0)
Education		
Bachelor’s degree or higher	3831 (67.3)	NA
Chronic conditions		
Hypertension	1578 (27.3)	27 (23.1)
High cholesterol	1160 (20.1)	12 (10.3)
Coronary heart disease	300 (5.2)	5 (4.3)
Angina, also called angina pectoris	281 (4.9)	1 (0.9)
Heart attack	264 (4.6)	0 (0.0)
Stroke	254 (4.4)	4 (3.5)
Asthma	889 (15.4)	5 (4.4)
Cancer or a malignancy of any kind	295 (5.1)	8 (6.9)
Diabetes	678 (9.1)	10 (8.7)
Chronic obstructive pulmonary disease, COPD, emphysema, or chronic bronchitis	293 (5.1)	3 (2.6)
Some form of arthritis, rheumatoid arthritis, gout, lupus, or fibromyalgia	728 (12.6)	11 (9.4)
Any type of anxiety disorder	1618 (28.1)	24 (20.5)
Any type of depression	2005 (34.7)	29 (24.8)
Chronic or seasonal allergies or sinus trouble	2112 (36.6)	31 (26.7)
Back pain	2307 (40.0)	29 (25.0)
Sciatica or radiating leg pain	808 (14.0)	11 (9.5)
Neck pain	1393 (24.2)	21 (18.1)
Trouble seeing, even when wearing glasses or contact lenses	817 (14.2)	6 (5.1)
Dermatitis or other chronic skin rash	637 (11.1)	11 (9.4)
Stomach trouble	1204 (20.9)	13 (11.1)
Trouble hearing, including deafness, in one or both ears	442 (7.7)	4 (3.4)
Trouble sleeping	2054 (35.6)	21 (18.3)
Last saw a doctor or other health professional about your health		
Within the past year	NA	58 (49.2)
How many times to a hospital emergency room during the last 12 months?		
≥ 1	NA	30 (25.4)
During the past 12 months, have you been hospitalized overnight?		
Yes	NA	19 (16.1)

*NA* not assessed

#### MTurk preference sample

We surveyed a second sample of MTurk respondents to elicit item pair preferences for measure finalization. The analytic sample included 124 respondents with demographic characteristics similar to the development sample: median age of 37 years, predominantly White (83%), non-Hispanic (95%), and male (63%). Rates of endorsement for chronic conditions ranged from 0% (heart attack) to 27% (allergies or sinus trouble). Nearly 75% of participants reported having seen a healthcare provider in the past two years (see Table 1).

#### Stakeholder panel

To ensure broad-based buy-in of the content of the new PROMIS profile measure, we consulted with a key stakeholder panel of individuals representing clinical care, PROMIS developers, researchers and adopters, and patient advocates (see Supplement Table S1).

All procedures were reviewed and approved by the research team’s institutional review board (RAND Human Subjects Research Committee FWA00003425; IRB00000051) and conform to the principles in the Declaration of Helsinki.

### Measures

#### Participant demographics

Surveys administered to the MTurk development and preference samples included questions about demographic characteristics and 22 health conditions. The preference sample was also asked how long it had been since last seeing a doctor or other health professional and their number of emergency room visits and hospital stays in the past year.

#### Candidate items for the short PROMIS profile

The development sample survey included 50 PROMIS items from four overlapping sources (see Tables 2 and S2) as candidates for the short PROMIS profile. The four sources include items assessing eight PROMIS domains (physical function, fatigue, sleep disturbance, pain interference, anxiety, depression, ability to participate in social roles and activities [social roles] and cognitive function—abilities [cognitive function]) and were selected based on discussions among the project team and PROMIS developers. Item sources 2 and 3 (described below) meet some of the criteria for a short PROMIS profile (brief, measure multiple domains) and thus contain attractive candidate items. However, these are custom forms and the sources have not been officially adopted and made available by PROMIS as unique stand-alone measures.

**Table 2:** Number of candidate items and source for PROMIS-16 by domain

Domain (abbreviation)	# of items	PROMIS-29 + 2	PROPr-14	UPMC16	SIGNAL
Physical function (PF)	8	x	x	x	
Ability to participate in social roles and activities (SOC)	6	x	x	x	x
Anxiety (ANX)	4	x		x	
Depression (DEP)	6	x	x	x	x
Sleep disturbance (SLP)	8	x	x	x	x
Pain interference (PI)	6	x	x	x	x
Cognitive function (CF)	5	x	x	x	x
Fatigue (FTG)	7	x	x	x	x

PROMIS-29 + 2 = Four items each to assess domains of physical function, fatigue, sleep disturbance, pain interference, anxiety, depression, and social roles, and two items to assess cognitive function; PROPr-14 = Two items each to assess domains of physical function, fatigue, sleep disturbance, pain interference, depression, social roles, and cognitive function; UPMC16 = Two items each used in routine clinical data collection in specialty ambulatory care clinics at UPMC to assess domains of physical function, fatigue, sleep disturbance, pain interference, anxiety, depression, social roles, and cognitive function; SIGNAL = One item each assessing fatigue, pain interference, depression, social roles, and cognitive function; two items assessing sleep (one sleep disturbance, one sleep-related impairment)

*Item Source #1: PROMIS-29* + *2 Profile* [3]. Four items each to assess domains of physical function, fatigue, sleep disturbance, pain interference, anxiety, depression, and social roles, and two items to assess cognitive function (30 items total, 17 unique to this source; as it is not scored with any of the eight target domains, the single pain intensity item was not a candidate for the profile composition).

*Item Source #2: PROPr initial valuation items (PROPr-14)* [11]*.* Two items each to assess domains of physical function, fatigue, sleep disturbance, pain interference, depression, social roles, and cognitive function (14 items total, 10 unique to this source).

*Item Source #3: University of Pittsburgh Medical Center (UPMC) (UPMC16)* [12]*.* Two PROMIS items each used in routine clinical data collection in specialty ambulatory care clinics at UPMC to assess domains of physical function, fatigue, sleep disturbance, pain interference, anxiety, depression, social roles, and cognitive function selected based on their strong psychometric properties and perceived clinical relevance (16 items total, 6 unique to this source).

*Item Source #4: PROMIS items having high ‘signal’ and/or being likely to be administered in the PROMIS Computer-Adaptive Testing (CAT) algorithm (SIGNAL).* One item each assessing fatigue, pain interference, depression, social roles, and cognitive function; two items assessing sleep (one sleep disturbance, one sleep-related impairment) (7 items total, 2 unique to this source).

PROMIS HRQOL domain scores were generated for all possible item pairs within the eight domains using established parameters from the PROMIS item banks (parameters for the sleep-related impairment item were generated based on calibration to the sleep disturbance items) and converted to the T-score metric (M = 50, SD = 10) per PROMIS convention. All domains except for sleep disturbance were centered on a general population mean of 50. The sleep disturbance domain used a combined general population and clinical sample for centering the T-score metric. Throughout the results section, item pairs are referred to as [*domain abbreviation_item1item2*] following the list in Table S2. As a gold standard, we generated the PROMIS-29 4-item domain scores and a 5-item cognitive function domain score using all the candidate cognitive function items. We use the term “gold standard” to evaluate how well the newly created short PROMIS Profile compares in psychometric properties to the longer established PROMIS-29 Profile measure.

#### Item pair preference questions

In addition to items assessing demographic characteristics, health conditions, and health utilization, preference sample respondents were presented with sets of two-item pair choices representing six of the eight PROMIS Profile domains (sleep and fatigue items were selected without preference sample input). Respondents were asked to “read the question pairs and use the radio buttons to indicate which pair they liked the best.” (See Supplement Fig. S1).

### Approach

The goal of the developmental approach, conducted in three phases, was to select the best item pair to represent each domain. In the first phase, we conducted an empirical evaluation of all candidate PROMIS items and item pairs using data from the MTurk development sample (*N* = 5775) to identify item pairs with relatively poor performance. This included basic descriptive information and performance of domain-specific T-scores for all item pairs relative to the gold standard (correlations with the gold standard and standardized mean differences from the gold standard with Cohen’s *d*) [13]. We also asked the stakeholder panel to select, for each HRQOL domain, the two items that ‘taken together, best reflect the domain’ based on item content. Ten of the thirteen stakeholders contributed initial ratings. We used the results from phase 1 to rule out several candidate pairs per domain.

In phase 2, we held our first stakeholder meeting in which we summarized the findings from phase 1, including the stakeholder preferences and discussed the remaining candidate pairs considering their content and psychometric information relative to the gold standard to agree on a reduced set of candidate pairs for further consideration. Item pair performance was presented graphically using item response theory (IRT)-based information curves [14–16]. These curves display information (presented on the y-axis) as a continuous function that varies according to the underlying domain score (presented on the x-axis). Estimates of precision (standard error and reliability) can be derived from information, and the presentation of multiple item pairs on a single plot effectively display their relative performance. Higher information magnitude reflects increased reliability and lower standard error.

Phase 3 included a second survey of the stakeholder panel and the MTurk preference sample to solicit their preferences between remaining candidate pairs for each domain. A total of nine stakeholders and 124 adult MTurk respondents provided preference ratings at this phase. We arrived at a proposed final PROMIS-16-item set, selected based on the preference ratings, and held a second stakeholder meeting to review the set’s basic descriptive statistics and obtain stakeholder approval for the final PROMIS-16 items.

## Results

### Phase 1

Across the eight HRQOL domains, empirical analyses revealed limited variability in the performance of the item pairs but did highlight some as performing better than others (see Table 3). In general, the T-score means and ranges showed values clustered around the population mean of 50, although anxiety and depression were slightly worse, whereas social role participation was slightly better. Correlations among items within each domain varied somewhat, with the largest ranges for the physical function and sleep disturbance domains. The average correlation among items was highest for pain interference and lowest for sleep disturbance. Item pair correlations with the gold standard were more consistent, although pairs composed of items from the PROMIS-29 + 2 were more highly correlated. A similar pattern was seen in the standardized mean differences of item pairs with the gold standard. Effect sizes for these mean differences tended to be small, although some exceeded 0.2 (small effect) within the physical function domain.

**Table 3:** Item pair performance summary by domain

Domain	N pairs	Mean T-score		Correlations among items in each domain		Correlations with gold standard		Mean difference from gold standard (Cohen’s d)	
		Average	Range	Average	Range	Average	Range	Average	Range
Physical function	28	49.4	47.6–51.7	0.57	0.28–0.71	0.87	0.69–0.96	0.05	− 0.17–0.33
Ability to participate in social roles and activities	15	52.6	51.7–53.3	0.74	0.68–0.79	0.94	0.89–0.97	− 0.06	− 0.16–0.01
Anxiety	6	53.9	52.9–54.8	0.70	0.67–0.77	0.95	0.94–0.96	− 0.04	− 0.15–0.06
Depression	15	52.6	51.6–53.8	0.71	0.63–0.79	0.92	0.83–0.97	− 0.05	− 0.14–0.07
Sleep disturbance	28	49.6	48.8–50.4	0.52	0.29–0.78	0.85	0.66–0.97	0.01	− 0.08–0.10
Pain interference	15	51.3	50.4–52.1	0.76	0.70–0.83	0.94	0.86–0.98	− 0.02	− 0.12–0.07
Cognitive function; abilities	10	50.6	49.5–52.1	0.56	0.44–0.64	0.91	0.82–0.94	− 0.03	− 0.16–0.13
Fatigue	21	49.6	47.9–51.3	0.68	0.46–0.81	0.92	0.82–0.97	− 0.03	− 0.20–0.15

The PROMIS-29 + 2 domain score was used as the gold standard for all domains except cognitive function which used a score based on the five candidate cognitive function items

Stakeholder preferences were quite varied for physical function, social roles, anxiety, and depression and somewhat more consistent for sleep disturbance, pain interference, cognitive function, and fatigue (see rightmost column of Supplement Table S2).

### Phase 2

**Fig. 1:**
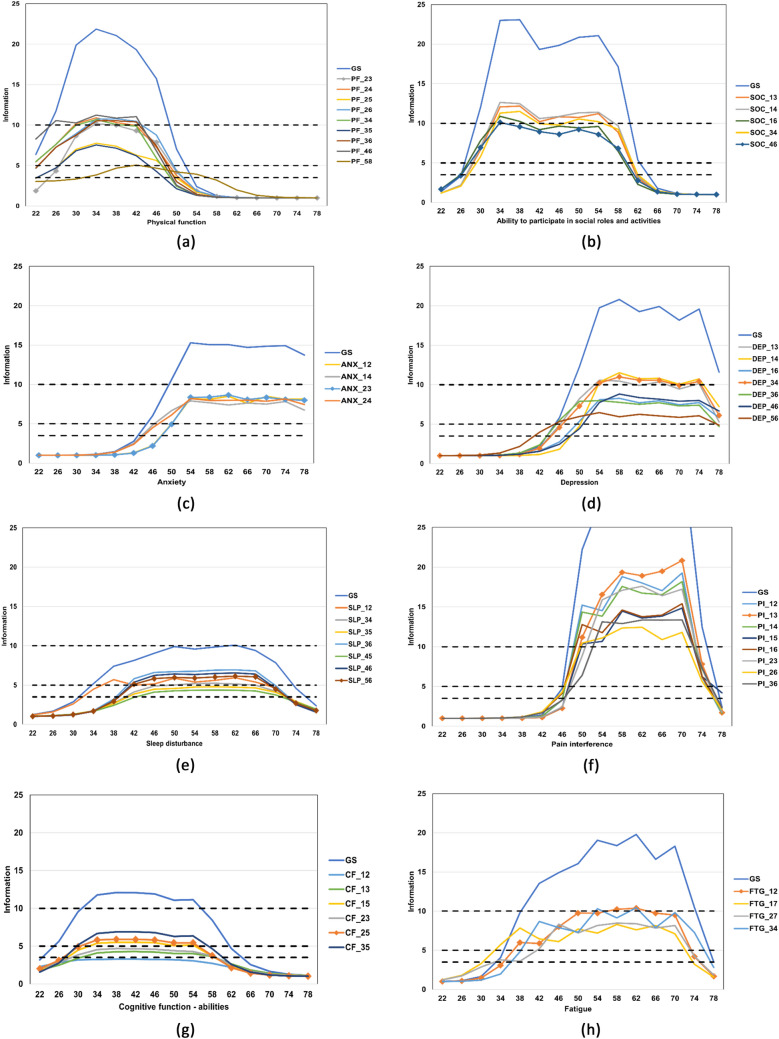
Gold standard (GS) and item pair information curves by domains of the PROMIS-16 presented to stakeholders. **a** Physical function (PF); **b** ability to participate in social roles and activities (SOC); **c** anxiety (ANX); **d** depression (DEP); **e** sleep disturbance (SLP); **f** pain interference (PI); **g** cognitive function—abilities (CF); **h** fatigue (FTG). Numbers following the domain abbreviations in the figure legend identify the specific item pair as listed in Supplement Table S2. SOC_16 and SOC_46 (social roles domain) were not presented in the first stakeholder meeting but were added to the figure after discussion. In each domain, the selected item pair has a diamond marker. The dashed lines indicate the cut-offs for reliability, with reliability of 0.90 at the upper line; 0.80 at the middle line; and 0.71 at the bottom line

We considered empirical IRT information functions and stakeholder ratings from phase 1, as well as IRT item parameters (thresholds and discrimination), to exclude some item pairs and prioritize others, reducing the number of pairs in each domain for further discussion during the stakeholder meeting. Fig. 1a–h displays IRT information curves for the remaining pairs plotted together with the gold standard for each domain and reveal variable degrees of precision across the domain score continua among the remaining set of item pairs for each domain.

During the discussion of each HRQOL domain, stakeholders considered the relative merits of item pairs that provided reasonable precision (reliability > 0.8) [15, 16] across a wide range of the T-score continuum, rejecting some item pairs based on content preferences and others based on format. For example, in the physical function domain, stakeholders noted that items in pair PF_26 use different formats and response options, mixing item stem introductions *Are you able…* with *Does your health now limit you…* and response options *Without any difficulty—Unable to do* with *Not at all—Cannot do*. In another example, stakeholders noted that the candidate items for social roles could be separated into two content groups, one representing more leisure or recreational roles (items 1, 4, and 5) and the other reflecting responsibility or work-related roles (items 2, 3, and 6) and recommended selecting an item pair representing these two aspects of social role participation. In this way, the stakeholders narrowed down the set of candidate pairs for all domains substantially during the meeting, reaching consensus on the final item pairs for sleep disturbance and fatigue. Following the meeting, the study team synthesized the stakeholder discussion points with the empirical evidence and item parameters and narrowed the set of remaining pairs to two pair options per domain.

### Phase 3

Preference ratings from the stakeholders and MTurk preference sample respondents were remarkably consistent and the final item pair for each HRQOL domain was selected based on these ratings (see Table 4). Stakeholders had no objections to the selected item set when presented with the psychometric performance of the 16 items at the second stakeholder meeting.

**Table 4:** Preference ratings by stakeholders and MTurk preference sample

Domain	Rated pairs	Selected pair	Stakeholders*	MTurk sample
Physical function	PF_23, PF_56	PF_23	5/9	83%
Ability to participate in social roles	SOC_16, SOC_46	SOC_46	5/9	56%
Anxiety	ANX_23, ANX_24	ANX_23	5/9	61%
Depression	DEP_34, DEP_35	DEP_34	5/9	51%
Sleep disturbance	NA	SLP_56	NA	NA
Pain interference	PI_12, PI_13	PI_13	6/9	58%
Cognitive function - abilities	CF_15, CF_25	CF_25	7/9	56%
Fatigue	NA	FTG_12	NA	NA

Nine out of 13 stakeholders provided ratings; final sleep disturbance and fatigue pairs were selected previously by stakeholders during the stakeholder meeting

*PF* physical function, *SOC* ability to participate in social roles and activities, *ANX* anxiety, *DEP* depression, *SLP* sleep disturbance, *PI* pain interference, *CF* cognitive function—abilities, *FTG* fatigue, numbers following domain abbreviations identify the specific item pair as listed in Supplement Table S2, *NA* not assessed

### PROMIS-16: item content and psychometric properties of HRQOL domain scores

The final version of the PROMIS-16 Profile contains 16 items measuring eight HRQOL domains with two items per domain. The measurement precision of the two-item domain scores is displayed in Fig. 1a–h, wherein a line with a marker depicts the selected item sets. The two-item sets provide acceptable precision across a moderate range of the score continuum for all domains. Although information curves for some domains fall below reliability of 0.7 at the low or high ends of the score continua, this tendency is also evident in the 4-item scales. The means of the domain T-scores ranged from 49.3 for fatigue to 54.8 for anxiety (see Table 5). Across the eight domains, there were moderate to strong correlations between items, ranging from 0.50 for cognitive function to 0.77 for anxiety and pain interference. All domains were highly correlated with the gold standard. This correlation exceeded 0.90 for all domains except sleep disturbance which correlated at 0.80. This result is expected given that the PROMIS-16 sleep disturbance domain does not share any items with the PROMIS-29, whereas other domains have some degree of item overlap. The standard mean differences of the final pairs with the gold standard were small, with five of the eight domains showing absolute effect sizes ≤ 0.060; absolute effect sizes for physical function (Cohen’s *d* = 0.11), ability to participate in social roles and activities (Cohen’s *d* = − 0.14), and cognitive function—abilities (Cohen’s *d* = − 0.16), while still considered trivial, exceeded 0.1. Table 6 shows the intercorrelations among domain scores for the PROMIS-16 (above diagonal) and the PROMIS-29 + 2 (below diagonal). The pattern and magnitude of relationships look similar across the two sources. Table 7 contains the item content, response options, and response frequencies for the PROMIS-16 by domain. In most cases, item response frequencies are distributed across the five response options, although the more extreme response options tend to have low endorsement rates. The table format and layout reflect the suggested format for administration. A version for administration is provided as Supplement Table S3. Because pairs of items with five response options produce a limited number of response patterns, the domain scoring of the PROMIS-16 is straightforward to document. Supplement Table S4 provides a scoring look-up table for the PROMIS-16, listing T-scores by domain for each item-pair response pattern.

**Table 5:** Item pair performance by domain for the 16-item PROMIS-16

Domain	Mean (SD) T-score	Correlation between items (95% CI)	Correlation with gold standard (95% CI)	Mean difference from gold standard (Cohen’s d; 95% CI)
Physical function	49.9 (7.6)	0.71 (0.69, 0.72)	0.92 (0.91, 0.92)	0.11 (0.08, 0.13)
Ability to participate in social roles and activities	51.9 (9.2)	0.72 (0.71, 0.73)	0.93 (0.93, 0.93)	− 0.14 (− 0.17, − 0.12)
Anxiety	54.8 (9.6)	0.77 (0.75, 0.78)	0.94 (0.93, 0.94)	0.06 (0.03, 0.09)
Depression	53.0 (9.8)	0.75 (0.74, 0.77)	0.97 (0.97, 0.97)	0.00 (− 0.03, 0.02)
Sleep disturbance	49.6 (8.4)	0.64 (0.62, 0.65)	0.80 (0.79, 0.81)	0.01 (− 0.01, 0.04)
Pain interference	52.0 (8.7)	0.77 (0.75, 0.78)	0.98 (0.98, 0.98)	0.06 (0.04, 0.09)
Cognitive function—abilities	49.5 (8.5)	0.50 (0.48, 0.52)	0.93 (0.92, 0.93)	− 0.16 (− 0.18, − 0.13)
Fatigue	49.3 (9.6)	0.70 (0.69, 0.71)	0.96 (0.96, 0.97)	− 0.06 (− 0.09, − 0.04)

The PROMIS-29 + 2 domain score was used as the gold standard for all domains except cognitive function which used a score based on the five candidate cognitive function items

**Table 6:** Correlations of the PROMIS-16 (above diagonal) and PROMIS-29 + 2 (below diagonal) domains

	PF	SOC	ANX	DEP	SLP	PI	CF	FTG
PF		0.54	− 0.38	− 0.37	− 0.35	− 0.63	0.33	− 0.42
SOC	0.64		− 0.64	− 0.63	− 0.59	− 0.67	0.48	− 0.65
ANX	− 0.43	− 0.66		0.77	0.56	0.50	− 0.48	0.65
DEP	− 0.43	− 0.66	0.82		0.58	0.47	− 0.45	0.69
SLP	− 0.30	− 0.49	0.52	0.53		0.49	− 0.38	0.64
PI	− 0.72	− 0.71	0.51	0.50	0.37		− 0.38	0.51
CF	0.33	0.39	− 0.37	− 0.37	− 0.31	− 0.31		− 0.41
FTG	− 0.47	− 0.68	0.70	0.71	0.61	0.54	− 0.30	

*PF* physical function, *SOC* ability to participate in social roles and activities, *ANX* anxiety, *DEP* depression, *SLP* sleep disturbance, *PI* pain interference, *CF* cognitive function—abilities, *FTG* fatigue

**Table 7:** PROMIS-16-item content and response frequencies (*N*, %) from MTurk development sample (*N* = 5775)

	Without any difficulty	With a little difficulty	With some difficulty	With much difficulty	Unable to do
**Physical function**					
Are you able to go up and down stairs at a normal pace?…	3524 (61)	1255 (22)	712 (12)	217 (4)	66 (1)
Are you able to go for a walk of at least 15 min?	4014 (70)	946 (16)	529 (9)	201 (4)	79 (1)

**Ability to participate in social roles and activities**	Never	Rarely	Sometimes	Usually	Always
I have trouble taking care of my regular personal responsibilities…	2409 (42)	1326 (23)	1301 (23)	515 (9)	187 (3)
I have trouble doing all of the activities with friends that I want to do…	2568 (45)	1330 (23)	1250 (22)	445 (8)	143 (3)

Anxiety (In the past 7 days…)	Never	Rarely	Sometimes	Usually	Always
I found it hard to focus on anything other than my anxiety…	2499 (43)	1444 (25)	1304 (23)	430 (8)	92 (2)
My worries overwhelmed me…	2294 (40)	1441 (25)	1338 (23)	534 (9)	164 (3)
**Depression (In the past 7 days…)**					
I felt depressed…	2203 (38)	1318 (23)	1371 (24)	641 (11)	236 (4)
I felt hopeless…	2805 (49)	1146 (20)	1170 (20)	479 (8)	167 (3)
**Sleep disturbance (In the past 7 days…)**					
I had trouble sleeping…	1593 (28)	1496 (26)	1625 (28)	750 (13)	285 (5)

	Not at all	A little bit	Somewhat	Quite a bit	Very much
I had problems during the day because of poor sleep…	2141 (37)	1746 (30)	1184 (21)	494 (9)	183 (3)
**Pain interference (In the past 7 days…)**					
How much did pain interfere with your day-to-day activities?….	2582 (45)	1613 (28)	978 (17)	429 (8)	134 (2)
How much did pain interfere with your ability to participate in social activities?….	3134 (55)	1188 (21)	836 (15)	413 (7)	160 (3)
**Cognitive function (In the past 7 days…)**					
I have been able to remember to do things, like take medicine or buy something I need…	377 (7)	627 (11)	1030 (18)	1460 (26)	2230 (39)
I have been able to think clearly without extra effort……	251 (4)	802 (14)	1182 (21)	1584 (28)	1893 (33)
**Fatigue (In the past 7 days…)**					
I feel fatigued…	1421 (25)	2023 (35)	1339 (23)	728 (13)	261 (5)
I have trouble starting things because I am tired …	1977 (34)	1828 (32)	1127 (20)	594 (10)	241 (4)

Response frequencies for some items sum to less than 5775 due to item-level missingness

## Discussion

This paper describes the development of a short 16-item HRQOL PROMIS Profile measure, the PROMIS-16, for use in research and clinical care. Items in the PROMIS-16 were selected from among a set of 50 candidate PROMIS items through rigorous empirical evaluation and consideration of stakeholder preferences. Because the PROMIS-16 uses existing PROMIS items, it has face validity, is straightforward to interpret, has multiple accessible administration options, and like other PROMIS scales will be easy to relate to other widely used measures both within and outside the PROMIS library. The use of only two items for each domain also enables easy access to pattern-based IRT scoring through the T-score look-up table provided as Supplement Table S4.

As the push to implement PRO measures (PROMs) in clinical care grows, the sustainability of these efforts requires careful consideration [17]. Implementation of PRO data collection in clinical practice requires measures that are short, relevant to the patient population being treated, rigorously developed and evaluated, easy to use and interpret, minimally disruptive to the clinical workflow, and have provider and patient buy-in [18, 19]. The PROMIS-16’s strong psychometric performance and estimation of clinically actionable HRQOL domain scores will likely lead to increased adoption of PROs in clinical practice. The reduction in patient burden relative to the longer profile measures is also beneficial for use in research, especially in studies that require the measurement of multiple outcomes or in which these are not the primary outcomes but are of interest to include as covariates. However, when HRQOL domain scores are a primary study outcome, longer scales may be preferable to provide adequate precision, especially at the extreme ends of score distributions. The PROMIS-16 may also prove useful for population health measurement and monitoring. In clinical care, the PROMIS-16 represents an ideal screening tool, which can help clinicians quickly identify distinct areas of concern that may require further assessment with longer, more targeted measures, and follow-up.

Preliminary evidence presented in this paper suggests that the eight HRQOL domain T-scores generated from the PROMIS-16 have strong psychometric properties, comparable in large part to those of the PROMIS-29 + 2 across a wide range of the score continuum. However, as can be seen in Fig. 1, the 4-item domain scores from the PROMIS-29 have better performance at the extremes. Further evaluation of the measure is needed and should include a more extensive evaluation of domain scores as well as evaluation of physical and mental summary scores and the overall utility score (PROPr).

There is a strong precedent for the viability and attractiveness of an ultra-short HRQOL Profile measure. When shorter versions of PROMs are used, they can result in higher acceptance and response rates and less missing data while having minimal impact on the psychometric performance compared to the long-form version of the PROM [20]. We made considerable effort to select items that cover a wide range of relevant content with adequate precision across the largest score range possible. However, the precision/brevity trade-off is challenging to balance, and there is an impact on psychometric performance that should be considered when using these shorter PROMs.

The strengths of longer measures are that they will have more precision, especially in the lower and upper ends of the score distribution, which is important for either discriminating among patients or examining change within an individual over time. In addition, more content from the HRQOL domain can be included with longer forms strengthening content validity. Thus, although the two-item T-scores represent valid mean estimates, there are many situations where more precision may be needed. For example, a study focused on depression as a primary outcome should include more than two items to assess that construct. Similarly, if used as a screener, responses to the two items that reach a level of clinical concern should trigger the administration of additional items and clinician probes to determine the severity of the problem and identify appropriate next steps. These limitations are particularly salient in the measurement of physical function where we elected to focus measurement with two items reflecting the mobility subdomain. PROMIS-16 users should be aware of the restricted content range of the physical function domain as it may be problematic to compare clinical populations experiencing physical limitations in different body areas [21].

In sum, the PROMIS-16 was selected to optimally balance measurement length and precision trade-offs, making it possible to assess eight core domains covering a broad range of physical and mental health aspects of HRQOL with minimal burden to respondents. Its availability will increase the inclusion of domain-specific HRQOL outcomes in clinical care and clinical and health-related research.

The results of this study should be considered with several limitations in mind. First, our development work was based on data from a single online sample of experienced survey takers who were predominantly White and non-Hispanic and relatively highly educated, thus this paper provides only preliminary evidence. However, our use of PROMIS items with established parameters mitigates this limitation considerably. Second, due to the homogeneity of empirical performance results, our selection was heavily influenced by item content, and we relied heavily on stakeholder input. Although this reliance on content preferences and stakeholder input may be seen as a limitation, it also points to the quality of the candidate PROMIS items. Further, our reliance on stakeholder input conveys their buy-in and will facilitate PROMIS-16 uptake.

This paper describes the development of the PROMIS-16 Profile, an ultra-short measure which generates eight domain-specific HRQOL scores for physical function, ability to participate in social roles and activities, anxiety, depression, sleep disturbance, pain interference, cognitive function, and fatigue with two items per domain. The inclusion of these eight domain scores in the PROMIS-16 makes it possible to generate physical and mental health summary scores following Hays et al. (2018) and the PROMIS-preference (PROPr) score, which are described in forthcoming manuscripts. The physical and mental health summary score derivation includes a sensitivity analysis to evaluate the impact of excluding the pain intensity item from the physical health summary score (because that item is not included in the PROMIS-16). Subsequent work will examine the correspondence of PROMIS-16 summary scores and global health (global-10) summary scores and establish a crosswalk between these two sets of summary scores. Although future work remains to establish the summary and preference scores and validate the domain score findings in an independent sample, preliminary results presented here indicate that the PROMIS-16, a short, rigorous HRQOL profile measure can be translated to domain-specific action and will be a useful tool for clinicians and researchers.

## Supplementary Information

Below is the link to the electronic supplementary material.

Supplementary file1 (PDF 252 KB)

Supplementary file2 (DOCX 46 KB)
