# Correction: Development of an ultra-short measure of eight domains of health-related quality of life for research and clinical care: the patient-reported outcomes measurement information system^Ⓡ^ PROMIS^Ⓡ^-16 profile

**DOI:** 10.1007/s11136-024-03762-5

**Published:** 2024-08-27

**Authors:** Maria Orlando Edelen, Chengbo Zeng, Ron D. Hays, Anthony Rodriguez, Janel Hanmer, Judy Baumhauer, Judy Cella, Bryce B. Reeve, Patricia M. Herman

**Affiliations:** 1 Patient Reported Outcomes, Value and Experience (PROVE) Center, Department of Surgery, Brigham and Women’s Hospital, Boston, MA USA; 2 RAND Corporation, Behavioral and Policy Sciences, 20 Park Plaza #920, Boston, MA USA; 3 Division of General Internal Medicine and Health Services Research, UCLA Department of Medicine, 20 Park Plaza #920, Los Angeles, CA USA; 4 Division of General Internal Medicine, University of Pittsburgh, Pittsburgh, PA USA; 5 School of Medicine and Dentistry, University of Rochester, Rochester, NY USA; 6 Feinberg School of Medicine, Northwestern University, Chicago, IL USA; 7 Department of Population Health Sciences, Duke University School of Medicine, Durham, NC USA; 8 Behavioral and Policy Sciences, RAND Corporation, 1776 Main Street, Santa Monica, CA USA

**Correction to: Quality of Life Research**

10.1007/s11136-023-03597-6

This correction addresses errors in table 7, S1 and S3, and makes a change to the footnote of table S4. The correction in table S1 addresses a misspelling of Dr. Amy Cizik’s name. This has been corrected. The errors in tables 7 and S3 are in the format and wording of the sleep questions. The sleep1 question is incorrectly specified as “I had problems during the day because of my sleep.” It should be specified as “I had problems during the day because of poor sleep”. This has been corrected. In addition, the “problems during the day” question should have response options “Not at all - very much” and the “trouble sleeping” question should have response options “Never – Always” thus the questions were reordered in corrected tables 7 and S3 to align with their correct response options. The footnote in table S4 was modified to alert users to the fact that questions sleep1 and sleep2 are presented in reverse order in table S3.

The correct version of table 7 and the supplementary information files are provided in this correction.

**Table 7:** PROMIS-16 item content and response frequencies (N, %) from MTurk development sample (*N* = 5775)

	Without any difficulty	With a little difficulty	With some difficulty	With much difficulty	Unable to do
Physical Function					
Are you able to go up and down stairs at a normal pace?…	3524 (61)	1255 (22)	712 (12)	217 (4)	66 (1)
Are you able to go for a walk of at least 15 min?	4014 (70)	946 (16)	529 (9)	201 (4)	79 (1)
**Ability to Participate in Social Roles and Activities**	**Never**	**Rarely**	**Sometimes**	**Usually**	**Always**
I have trouble taking care of my regular personal responsibilities……………	2409 (42)	1326 (23)	1301 (23)	515 (9)	187 (3)
I have trouble doing all of the activities with friends that I want to do…………….	2568 (45)	1330 (23)	1250 (22)	445 (8)	143 (3)
**Anxiety (In the past 7 days…)**	**Never**	**Rarely**	**Sometimes**	**Often**	**Always**
I found it hard to focus on anything other than my anxiety…	2499 (43)	1444 (25)	1304 (23)	430 (8)	92 (2)
My worries overwhelmed me…………….	2294 (40)	1441 (25)	1338 (23)	534 (9)	164 (3)
**Depression (In the past 7 days…)**					
I felt depressed…………………….	2203 (38)	1318 (23)	1371 (24)	641 (11)	236 (4)
I felt hopeless……………	2805 (49)	1146 (20)	1170 (20)	479 (8)	167 (3)
**Sleep Disturbance (In the past 7 days…)**					
I had trouble sleeping…………….	1593 (28)	1496 (26)	1625 (28)	750 (13)	285 (5)
	**Not at all**	**A little bit**	**Somewhat**	**Quite a bit**	**Very much**
I had problems during the day because of poor sleep…………	2141 (37)	1746 (30)	1184 (21)	494 (9)	183 (3)
**Pain Interference (In the past 7 days…)**					
How much did pain interfere with your day-to-day activities?………….	2582 (45)	1613 (28)	978 (17)	429 (8)	134 (2)
How much did pain interfere with your ability to participate in social activities?…….	3134 (55)	1188 (21)	836 (15)	413 (7)	160 (3)
**Cognitive Function (In the past 7 days…**					
I have been able to remember to do things, like take medicine or buy something I need….	377 (7)	627 (11)	1030 (18)	1460 (26)	2230 (39)
I have been able to think clearly without extra effort……	251 (4)	802 (14)	1182 (21)	1584 (28)	1893 (33)
**Fatigue (In the past 7 days…)**					
I feel fatigued………….	1421 (25)	2023 (35)	1339 (23)	728 (13)	261 (5)
I have trouble starting things because I am tired …	1977 (34)	1828 (32)	1127 (20)	594 (10)	241 (4)

Response frequencies for some items sum to less than 5775 due to item-level missingness

The original article has been corrected.

## Electronic supplementary material

Below is the link to the electronic supplementary material.

Supplementary Material 1

