# Supplementary material for: Development of an ultra-short measure of eight domains of health-related quality of life for research and clinical care: the patient-reported outcomes measurement information system® PROMIS®-16 profile
Source: Qual Life Res. 2024 Feb 6;34(1):3–15. doi: 10.1007/s11136-023-03597-6 (PMC11800902; doi:10.1007/s11136-023-03597-6)
Supplement: Supplementary file 1 — Supplementary file1 (PDF 252 KB) [file 11136_2023_3597_MOESM1_ESM.pdf]

Table S1. Stakeholder Panel Members

Name	Affiliation	Expertise
John L. Adams, PhD	Kaiser Permanente Center for Effectiveness and Safety Research	Statistician; use of PROMs for performance assessment
Judy Baumhauer, MD, MPH	University of Rochester School of Medicine and Dentistry	Use of PROMIS in clinical practice
David Cella, PhD	Northwestern University, Feinberg School of Medicine	PROMIS Steering Committee Chair, PROMIS Statistical Coordinating Center PI
Penney Cowan	American Chronic Pain Association	Patient advocacy, pain management
Amy Cizik, PhD	University of Utah, Health System Innovation and Research	Use of PROMIS in clinical research and practice
David Feeny, PhD	McMaster University	Health economist, PROPr, HUI, utility measurement
Rachel Hess, MD, MS	University of Utah, Health System Innovation and Research	Health services research, patient-centered outcomes
Kurt Kroenke, MD, MACP	Regenstrief Institute; Indiana University School of Medicine	PROs in clinical care
Jacob Lippa, MPH	Providence	Clinical analytics, PRO data collection and storage
Bryce B. Reeve, PhD	Duke University School of Medicine, Department of Population Health Sciences	Item response theory; PROMIS, health measurement
Rachel Sisodia, MD	Mass General Brigham	PROs in clinical care
Joel Tsevat, MD, MPH	Dell Medical School, University of Texas at Austin	HRQoL research, PRO data collection
Albert Wu, MD, MPH	Johns Hopkins Bloomberg School of Public Health	Patient outcomes and quality of care

Table S2. PROMIS-16 Candidate Items by Source with Stakeholder Ratings

Item Stem by Domain	Source	N selected in initial stakeholder rating
**Physical Function (PF; 8 total)**		
1. Are you able to do chores such as vacuuming or yard work?	PROMIS 29+2	0
2. Are you able to go up and down stairs at a normal pace?	PROMIS 29+2	4
3. Are you able to go for a walk of at least 15 minutes?	PROMIS 29+2	5
4. Are you able to run errands and shop?	PROMIS 29+2	1
5. Does your health now limit you in bathing or dressing yourself?	UPMC 16	5
6. Does your health now limit you in doing moderate work around the house like vacuuming, sweeping floors or carrying in groceries?	UPMC 16	4
7. Are you able to dress yourself, including tying shoelaces and buttoning up your clothes?	PROPr 14	1
8. Are you able to run 100 yards (100 m)?	PROPr 14	0
**Ability to Participate in Social Roles and Activities (SOC; 6 total)**		
1. I have trouble doing all of my regular leisure activities with others	PROMIS-29+2, SIGNAL	4
2. I have trouble doing all of the family activities that I want to do	PROMIS-29+2	3
3. I have trouble doing all of my usual work (include work at home)	PROMIS-29+2	5
4. I have trouble doing all of the activities with friends that I want to do	PROMIS-29+2	4
5. I have trouble participating in recreational activities with others	PROPr 14, UPMC 16	1
6. I have trouble taking care of my regular personal responsibilities	PROPr 14, UPMC 16	3
**Anxiety (ANX; 4 total)**		
1. I felt fearful	PROMIS-29+2	5
2. I found it hard to focus on anything other than my anxiety	PROMIS-29+2	7
3. My worries overwhelmed me	PROMIS-29+2, UPMC 16	6
4. I felt uneasy	PROMIS-29+2, UMPC 16	2
**Depression (DEP; 6 total)**		
1. I felt worthless	PROMIS-29+2	0
2. I felt helpless	PROMIS-29+2	3

3. I felt depressed	PROMIS-29+2, UPMC 16, SIGNAL	6
4. I felt hopeless	PROMIS-29+2, UPMC 16	5
5. I felt unhappy	PROPr 14	2
6. I felt that nothing was interesting	PROPr 14	4
**Sleep Disturbance (SLP; 8 total)**		
1. My sleep quality was	PROMIS-29+2	1
2. My sleep was refreshing	PROMIS-29+2, UPMC	1
3. I had a problem with my sleep	PROMIS-29+2, SIGNAL	2
4. I had difficulty falling asleep	PROMIS-29+2	1
5. I had problems during the day because of poor sleep.	SIGNAL	9
6. I had trouble sleeping	UPMC 16	5
7. I got enough sleep	PROPr 14	0
8. I woke up too early and could not fall back to sleep	PROPr 14	1
**Pain Interference (PI; 6 total)**		
1. How much did pain interfere with your day to day activities	PROMIS-29+2	9
2. How much did pain interfere with work around the home?	PROMIS-29+2, UPMC 16, SIGNAL	1
3. How much did pain interfere with your ability to participate in social activities	PROMIS-29+2, UMPC 16	0
4. How much did pain interfere with your household chores?	PROMIS-29+2	0
5. How often was your pain so severe you could think of nothing else?	PROPr 14	1
6. How often was pain distressing to you?	PROPr 14	2
**Cognitive Function (CF; 5 total)**		
1. I have been able to concentrate	PROMIS-29+2, PROPr 14	6
2. I have been able to remember to do things, like take medicine or buy something I needed	PROMIS-29+2, PROPr 14	7
3. My memory has been as good as usual....	UPMC 16	2
4. I have been able to focus my attention.....	UPMC 16	3
5. I have been able to think clearly without extra effort	SIGNAL	2
**Fatigue (FTG; 7 total)**		
1. I feel fatigued	PROMIS-29+2, SIGNAL	4
2. I have trouble starting things because I am tired	PROMIS-29+2	6
3. How run-down did you feel on average?	PROMIS-29+2	1
4. How fatigued were you on average?	PROMIS-29+2, UPMC 16	2
5. How often were you bothered by your	UPMC 16	2

fatigue?		
6. How often were you too tired to take a bath or shower?	PROPr 14	2
7. How often did you feel tired?	PROPr 14	3

PROMIS-29+2 = Four items each to assess domains of physical function, fatigue, sleep disturbance, pain interference, anxiety, depression, and social roles, and two items to assess cognitive function; PROPr-14 = Two items each to assess domains of physical function, fatigue, sleep disturbance, pain interference, depression, social roles, and cognitive function; UPMC16= Two items each used in routine clinical data collection in specialty ambulatory care clinics at UPMC to assess domains of physical function, fatigue, sleep disturbance, pain interference, anxiety, depression, social roles, and cognitive function; SIGNAL= One item each assessing fatigue, pain interference, depression, social roles and cognitive function; two items assessing sleep (one sleep disturbance, one sleep-related impairment)

Table S3. PROMIS-16 administration form

Labels		Without any difficulty	With a little difficulty	With some difficulty	With much difficulty	Unable to do
**Physical Function**						
PF1 (PFA21)	Are you able to go up and down stairs at a normal pace?...	☐ 5	☐ 4	☐ 3	☐ 2	☐ 1
PF2 (PFA23)	Are you able to go for a walk of at least 15 minutes?	☐ 5	☐ 4	☐ 3	☐ 2	☐ 1
**Ability to Participate in Social Roles and Activities**		**Never**	**Rarely**	**Sometimes**	**Usually**	**Always**
SOC1 (SRPPER31_CaPS)	I have trouble taking care of my regular personal responsibilities..............	☐ 5	☐ 4	☐ 3	☐ 2	☐ 1
SOC2 (SRPPER46_CaPS)	I have trouble doing all of the activities with friends that I want to do…………….	☐ 5	☐ 4	☐ 3	☐ 2	☐ 1
**Anxiety (In the past 7 days…)**		**Never**	**Rarely**	**Sometimes**	**Often**	**Always**
ANX1 (EDANX40)	I found it hard to focus on anything other than my anxiety………………	☐ 1	☐ 2	☐ 3	☐ 4	☐ 5
ANX2 (EDANX41)	My worries overwhelmed me…………….	☐ 1	☐ 2	☐ 3	☐ 4	☐ 5
**Depression (In the past 7 days…)**						
DEP1 (EDDEP29)	I felt depressed…………………….	☐ 1	☐ 2	☐ 3	☐ 4	☐ 5
DEP2 (EDDEP41)	I felt hopeless…………..	☐ 1	☐ 2	☐ 3	☐ 4	☐ 5
**Sleep Disturbance (In the past 7 days…)**						
SLP2 (SLEEP90)	I had trouble sleeping……………	☐ 1	☐ 2	☐ 3	☐ 4	☐ 5
		**Not at all**	**A little bit**	**Somewhat**	**Quite a bit**	**Very much**
SLP1 (SLEEP25)	I had problems during the day because of poor sleep………………….	☐ 1	☐ 2	☐ 3	☐ 4	☐ 5
**Pain Interference (In the past 7 days…)**						
PI1 (PAININ9)	How much did pain interfere with your day-to-day activities?.............	☐ 1	☐ 2	☐ 3	☐ 4	☐ 5
PI2 (PAININ31)	How much did pain interfere with your ability to participate in social activities?........	☐ 1	☐ 2	☐ 3	☐ 4	☐ 5
**Cognitive Function (In the past 7 days…**						
COG1 (PC27)	I have been able to remember to do things, like take medicine or buy something I need….	☐ 1	☐ 2	☐ 3	☐ 4	☐ 5
COG2 (PC-CaPS3R)	I have been able to think clearly without extra effort……	☐ 1	☐ 2	☐ 3	☐ 4	☐ 5
**Fatigue (In the past 7 days…)**						
FTG1 (HI7)	I feel fatigued…………	☐ 1	☐ 2	☐ 3	☐ 4	☐ 5
FTG2 (AN3)	I have trouble starting things because I am tired …	☐ 1	☐ 2	☐ 3	☐ 4	☐ 5

Table S4. IRT T-Scores by Domain for Item Pair Response Patterns in the PROMIS-16 Profile

Item 1	Item 2	PF	SOC	ANX	DEP	SLP	PI	CF	FTG
1	1	27.45	28.32	43.45	41.67	37.64	42.89	28.64	35.1
1	2	31.81	32.61	52.68	51.63	45.78	52.37	35.43	43.84
1	3	33.8	35.53	55.61	53.84	50.74	54.31	40.05	47.64
1	4	36.28	39.11	57.73	55.85	55.36	55.53	44.99	50.15
1	5	40.02	42.13	58.81	56.91	59.37	56.26	51.31	51.85
2	1	32.18	34.81	53.32	50.7	43.28	52.75	32.31	43.31
2	2	34.25	36.52	57.05	55.04	48.63	56.07	37.11	48.56
2	3	35.87	39.04	60.04	57.45	53.18	58.53	41.19	52.31
2	4	38.04	42.16	62.69	59.77	57.48	60.34	45.77	54.98
2	5	41.49	45.14	64.38	61.13	61.41	61.59	51.8	56.95
3	1	34.7	39.38	56.63	54.68	45.5	56.52	35	48.79
3	2	36.34	40.55	60.38	58.27	50.81	59.2	39.16	52.72
3	3	37.83	43.05	63.76	61.21	55.47	61.55	42.94	56.3
3	4	39.88	46.28	67.06	64.18	59.73	63.74	47.25	59.4
3	5	43.26	49.58	69.77	66.24	63.87	65.58	52.96	61.98
4	1	37.82	45.27	58.92	57.87	46.69	58.97	37.65	52.33
4	2	39.11	45.89	63.05	61.38	52.29	61.75	41.51	56.35
4	3	40.46	47.69	67.03	64.75	57.33	64.22	45.17	59.87
4	4	42.47	50.58	70.92	68.24	61.81	66.71	49.46	63.32
4	5	46.27	54.22	74.53	71.35	66.53	69.19	55.21	66.67
5	1	42.25	50.64	59.87	59.65	47.24	60.97	39.89	54.49
5	2	43.2	51.03	64.35	63.6	53.11	63.98	43.77	58.92
5	3	44.33	52.37	68.99	67.76	58.65	66.76	47.57	62.94
5	4	46.25	55.02	73.94	71.98	63.66	69.69	52.3	67.06
5	5	56.08	62.12	79.48	77.03	70.48	74.41	61.03	72.55

PF: Physical function, items 1 and 2 correspond to PROMIS item labels PFA21 and PFA23 in Table S3 respectively; SOC: Ability to participate in social roles and activities, items 1 and 2 correspond to PROMIS item labels SRPPER31_CaPS and SRPPER46_CaPS in Table S3 respectively; ANX: Anxiety, items 1 and 2 correspond to PROMIS item labels EDANX40 and EDANX41 in Table S3 respectively; DEP: Depression, items 1 and 2 correspond to PROMIS item labels EDDEP29 and EDDEP41 in Table S3 respectively; SLP: Sleep disturbance, items 1 and 2 correspond to PROMIS item labels SLEEP25 and SLEEP90 in Table S3 respectively. NOTE that these items are presented in Table S3 in reverse order; PI: Pain interference, items 1 and 2 correspond to PROMIS item labels PAININ9 and PAININ31 in Table S3 respectively; CF: Cognitive function – abilities, items 1 and 2 correspond to PROMIS item labels PC27 and PC-CaPS3R in Table S3 respectively; FTG: Fatigue, items 1 and 2 correspond to PROMIS item labels HI7 and AN3 in Table S3 respectively.
