# Supplementary material for: Development of an ultra-short measure of eight domains of health-related quality of life for research and clinical care: the patient-reported outcomes measurement information system® PROMIS®-16 profile
Source: Qual Life Res. 2024 Feb 6;34(1):3–15. doi: 10.1007/s11136-023-03597-6 (PMC11800902; doi:10.1007/s11136-023-03597-6)
Supplement: Supplementary file 2 — Supplementary file2 (DOCX 46 KB) [file 11136_2023_3597_MOESM2_ESM.docx]

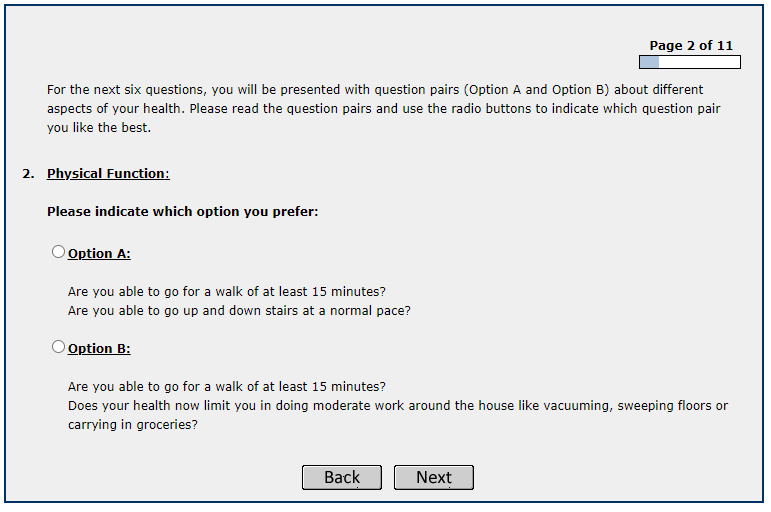


*Figure S1. Screen shot of first question on final preference survey administered to stakeholders and MTurk preference sample*
